# Antiseptics

**Published:** 1889-05

**Authors:** 


ARTICLE IV.

### ANTISEPTICS.

Dr. Black said that the antiseptic value of a drug is best expressed by its range of effective work. This range of value is found in the difference between the saturated solution, or that of concentration that may be found injurious to the tissues, and the greatest dilution that inhibits the development of micro-organisms. Those essential oils that are not too irritating have an extension of range in their use in emulsion, or in substance. Also many drugs have, in greater dilution than that which actually inhibits, a range of restraint that is useful.

The essential oils are growing in favor, but there is a lack of exact knowledge concerning their peculiarities. Many that are held in high esteem are not antiseptic at all, while others that are scarcely in use have very valuable antiseptic qualities. His experiments prove conclusively that iodoform has no antiseptic value, micro-organisms developing in the undissolved powder in the bottom of the test tubes used in the experiments. This also occurred in the emulsion of the oils of jaceput, thyme, wintergreen, and several other of the esteemed essential oils, among the inhibiting oils being the oils of cassia, cloves, cinnamon, mustard, peppermint, sassafras and others. The great difficulty in the use of the essential oils lies in their uncertain qualities, being usually prepared in the locality where the plant grows by unscientific workmen with probably a careless mingling of different parts of the plant possessing very different qualities. As their value becomes known we shall probably be able to secure their preparation in a more worthy manner.

In the experiments of Dr. Black peptonized beef broth, infected with his own saliva as far as possible under the same conditions, was used as the culture medium, the tubes graduated to secure accurate measurements, and kept five days in an incubating oven at a temperature of 99°, unless growths occurred earlier. The oils were tested both in substance and in solution. The aqueous solutions were made by violently shaking together, placing in the oven twelve hours, shaking again, and after a second twelve hours in the oven repeatedly filtered until perfectly clear. Every precaution was taken to secure exactness, every experiment being repeated until no doubt was left as to the result. One fact to be borne in mind is that the greater the range of inhibitory value the greater the toxic effects; if not poisonous their range of value is very short. Thus eucalyptus is very feeble, only a saturated solution being effective, but at the same time it is perfectly safe, having no toxic effects even by absorption. Bichloride of mercury which on the other hand has the greatest range of value, is also the most poisonous. Oil of cassia holds its own in comparison with carbolic acid, boracic acid, or resorcin. The pure oil blisters the skin, healing, however, perfectly beneath the blister; in emulsion, except in very large wound surfaces, it gives excellent results in preventing pus formation. It can be largely diluted with bland oils and give good results—"1—2—3" or

| | | |
|---|---|---|
| Carbolic acid | - - - | 1 |
| Oil of cassia | - - - | 2 |
| Oil of wintergreen | - - | 3 |

Will be found excellent, having an antiseptic range greater than carbolic acid alone, without its evil effects.

Antiseptics are used in three forms: in water for washings, the oil, and in the powder. Washings cannot be made continuous, therefore limited in usefulness in dental practice. Solutions in peroxide of hydrogen have a greater value than in water, as we have the added effect of the mechanical liberation of oxygen carrying the effects into the most remote parts. The oils are more effective from their diffusibility and remaining in position longer, doing their work continuously. They are especially applicable in the treatment of root canals where they will do good for many days together. Oil of cloves and cinnamon are less distasteful than some others. Mustard is too irritating, but is very valuable as a stimulant and is the most diffusible of all the oils. An emulsion of any of the oils may be made by shaking violently in a bottle with water and passing through a syringe a number of times.

Boracic acid stands at the head of the list for use in powder, as a dry antiseptic dressing. Hydronaphthol may be used in the same way but is not so well borne by the tissues and has less antiseptic value. Dry powder dressings are liable to cake and form an arch under which septic conditions may exist; this must be guarded against. A combination of oil and dry dressing gives enhanced value to both.

The fourth form, more rarely employed, is that of hypodermic injections where as in stumps, tumors, erysipeles, gangrene, etc., it is desirable that the tissues should be injected with the remedy. Dr. Black states that he had once prevented the amputation of a gangrenous stump higher up by injecting the tissues very completely with salycilic acid. It sloughed off with no recurrence of gangrene. It is found that liquids diffuse very slowly through contracted openings—as of abscesses and sinuses—hence the value of peroxide of hydrogen, and from this fact also we learn that the action of medicaments in a root canal will not extend beyond the apex unless forced through when applied.—*Dental Register*, Chicago Meeting, reported by Mrs. M. W. J.

